# Refining Liposuction Safety and Efficacy: Surgeon Learning-Curve Analysis With Helium-Based Plasma Integration

**DOI:** 10.1093/asjof/ojae063

**Published:** 2024-08-23

**Authors:** Paul G Ruff, Allison Martinez, Nicholas Burpee

## Abstract

**Background:**

The introduction of medical advancements requires ongoing critical evaluation of clinical practice and patient outcomes to improve results and safety. Since the development of minimally invasive, energy-based devices, this process has been occurring throughout the field of aesthetic medicine.

**Objectives:**

To collect retrospective procedure and safety data of liposuction procedures with or without adjunct utilization of a helium-based plasma device, compare 3 groups, and delineate the learning curve.

**Methods:**

A retrospective chart review at a single site included healthy patients ≥18 years of age treated by the principal investigator (PI). A total of 50 patients had an ultrasonic-assisted liposuction procedure, 50 patients had a liposuction procedure with the utilization of the helium-based plasma device, and 50 of the PI's most recent patients had a liposuction procedure with the utilization of the helium-based plasma device. All patients had at least 6 months of documented postoperative follow-up care.

**Results:**

Totally, 150 patients were enrolled in the study. Most patients had multiple body areas treated, primarily hips and abdomen. Treatment settings varied, with significant relationships found between pain and treatment groups (*P* = .013). No serious or unexpected adverse events (AEs) were reported, and all AE resolved before the final follow-up.

**Conclusions:**

The data collected support that patient outcomes and safety improve with continued use of the helium-based plasma device by the PI. The data also support the use of a helium-based plasma device as safe when used in combination with liposuction procedures.

**Level of Evidence: 4 (Therapeutic):**

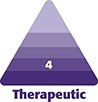

Minimally invasive devices that deliver thermal energy in the same subcutaneous tissue planes in which liposuction is performed have achieved the appropriate balance between excisional and noninvasive procedures. One study showed an average skin surface area reduction of 22% and 17% at the 1- and 3-month follow-up visits, respectively, and an average of 26% skin tightening at 3 months when utilizing laser-assisted liposuction (LAL) to address abdominal skin laxity.^1^ In 2008, radiofrequency (RF) devices were introduced and have demonstrated efficacy in multiple body areas including the abdomen,^2,3^ the arms,^4,5^ and the face and neck.^6,7^ One of the main challenges associated with the use of these minimally invasive devices to address skin laxity is the balance that must be achieved between heating the internal tissues enough to achieve the desired tissue/collagen contraction while still maintaining safe external tissue temperatures.

In 2016, a minimally invasive helium-based plasma device (Renuvion; Apyx Medical, Clearwater, FL) was first used for subdermal tissue heating to reduce skin laxity.^8^ Helium gas is passed over an electrode handpiece energized by RF waves, generating helium plasma. Heat is produced during the production of plasma itself through the ionization and rapid neutralization of helium atoms. Some of the RF energy is conducted from the electrode to the patient through the plasma beam, passing current through the resistance of the tissue, a process known as Joule heating. Since its introduction to his practice, the author has taken his understanding of the technology to improve the safety and efficacy of this adjunct procedure. His efforts contributed to the helium-based plasma device being awarded 510k clearance by the US Food and Drug Administration in July 2022, expanding its indication for the device to include subcutaneous dermatological and aesthetic procedures to improve the appearance of loose skin in the neck and submental region, as well as in April 2023 with the expanded indication of coagulation of subcutaneous soft tissues following liposuction for aesthetic body contouring.

To further these advancements, the purpose of this study was to collect real-world, retrospective procedure and safety data of liposuction procedures performed with or without the helium-based plasma device used as an adjunct procedure.

## METHODS

A retrospective chart review was conducted with a waiver of consent granted by Sterling IRB. The electronic medical records at a single site were reviewed to identify patients meeting the eligibility criteria. The eligible patients comprised 150 healthy patients ≥18 years of age who had undergone a liposuction procedure with or without the adjunct utilization of the helium-based plasma system that the principal investigator (PI) treated. Furthermore, the patients were classified into the following 3 groups: (1) 50 of the PI's patients who had an ultrasonic-assisted liposuction (UAL) procedure before the PI's utilization of the helium-based plasma system; (2) 50 of the PI's first few patients who had a UAL procedure with the utilization of the helium-based plasma system; and (3) 50 of the PI's most recent patients who had a UAL procedure with the utilization of the helium-based plasma system. The surgical cases included in this retrospective chart review were performed by the PI in sequential order. Cases in Group 1 were performed between April 21, 2011, and December 21, 2017; in Group 2 were performed between December 6, 2017, and December 18, 2018; and in Group 3 were performed between December 29, 2020, and December 7, 2021.

All patients were seen per the site standard of care for posttreatment follow-up and had a minimum of 6 months of documented postoperative follow-up care. Routine follow-up appointments are typically scheduled 1 day, 2 weeks, 6 weeks, 3 months, 6 months, and 1 year following surgery unless there are issues, such as seroma or prolonged edema, that would necessitate more frequent follow-up care. Where available, data on de-identified demographics, surgical variables/techniques, treatment settings data, length of follow-up, postoperative expected treatment effects, and adverse events (AEs)/complications through follow-up were collected. Expected treatment effect is defined as any typical treatment side effect of the procedure of mild-to-moderate severity and lasting up to a typical maximum duration. An AE is defined as any new medical problem, or exacerbation of an existing problem, experienced by the patient postoperatively, whether or not it is considered a procedure- or device-related issue by the investigator. Each AE was assessed for its seriousness and classified as serious if it met the following criteria: death was an outcome of the AE, the patient was at substantial risk of dying at the time of the AE, admission to the hospital or prolonged hospitalization was the result of the AE, the AE resulted in a substantial disruption of a person's ability to conduct normal life functions, exposure to a medical product before the conception may have resulted in an adverse outcome in the child, medical or surgical intervention was necessary to preclude permanent impairment of body function or prevent permanent damage to body structure, or the event does not fit the other outcomes but may jeopardize the patient and may require intervention to prevent one of the other outcomes.

All procedures were performed utilizing a 1:1 (50 cc of 2% Lidocaine and 0.5 cc of 1 mg/mL Epinephrine [1:1000]) tumescent infiltrate mixture and undermining by ultrasound-assisted method. During treatment, emulsified fluids and tissues were aspirated. The standard power setting for UAL was 70% in V mode. The volume of aspirate, where available, for all body areas treated, was recorded for each patient. Many patients had multiple (>1) body areas treated with UAL during the same procedure. The helium-based plasma device portion of the procedure was performed after any liposuction, implantation, or excisional aspect of the procedure. Standard pretreatment infusion of the planned area is performed first (mitrally) with an anesthetic wetting solution. This is followed by tunneling with an ultrasonic probe. Finally, treatment of the subdermal/subcutaneous space is performed with the helium-plasma RF handpiece size 15 or 27cm utilizing 2 to 7 passes. Each pass consists of multiple strokes spaced 1 to 2 cm apart, and passes are performed in multiple layers to ensure treatment of the 3-dimensional (3D) architecture (Video). Settings for the helium-plasma RF handpiece consisted of liters per minute (LPM) of helium ranging between 1.5 and 4 LPM and power ranging from 60% to 85%.

All patients were attempted to be contacted for the prospective portion of this retrospective study. For patients who were successfully contacted and agreed to participate, phone consent was obtained, as approved by Sterling IRB, before the participation in the patient satisfaction survey.

De-identified data were summarized in the descriptive statistics produced by Technomics Research LLC (Minneapolis, MN) and provided to the site. The site ran additional statistics (analysis of variance [ANOVA] test and χ^2^ tests) on data from patient demographics, expected treatment effects, and AEs.

## RESULTS

A total of 150 charts were reviewed for this study, including 36 males (24%, *n* = 36) and 114 females (76%, *n* = 114), with an average age of 47 years (range, 19-76 years) and an average BMI of 26.0 kg/m^2^ (range, 17.4-41.6 kg/m^2^; Table 1). All patients were treated with UAL across a total of 472 body areas. Among these patients, 100 were treated with the helium-based plasma system in 299 body areas as an adjunct procedure to UAL.

**Table 1.** Patient Demographics

	Group 1 (*n* = 50)	Group 2 (*n* = 50)	Group 3 (*n* = 50)	Total (*n* = 150)
Age (years)				
Mean ± SD	44.6 ± 12.4	48.6 ± 11.2	47.0 ± 11.6	46.8 ± 11.8
Median	42.0	50.0	48.0	47.0
Min, Max	(19.0, 70.0)	(24.0, 75.0)	(21.0, 76.0)	(19.0, 76.0)
Sex				
Female	72.0% (36/50)	76.0% (38/50)	80.0% (40/50)	76.0% (114/150)
Male	28.0% (14/50)	24.0% (12/50)	20.0% (10/50)	24.0% (36/150)
BMI				
Mean ± SD	27.5 ± 4.4	24.9 ± 4.0	26.0 ± 3.7	26.0 ± 4.1
Median	27.0	24.7	26.2	25.8
Min, Max	(22.2, 39.4)	(17.4, 41.6)	(18.3, 36.3)	(17.4, 41.6)

SD, standard deviation.

**Figure 1.**
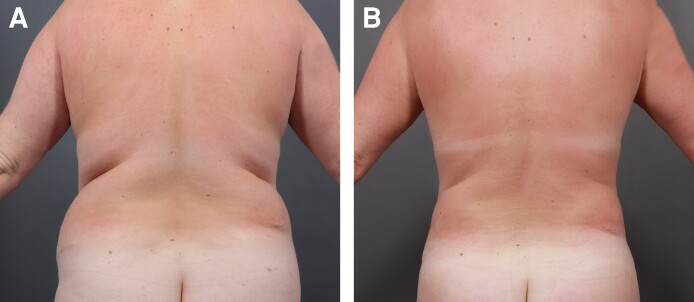
Results from an ultrasonic-assisted liposuction procedure without the utilization of a helium-based plasma system. A 35-year-old female (A) before and (B) after receiving an ultrasonic-assisted liposuction procedure without the utilization of a helium-based plasma system. After image was taken 1 year postoperatively.

**Figure 2.**
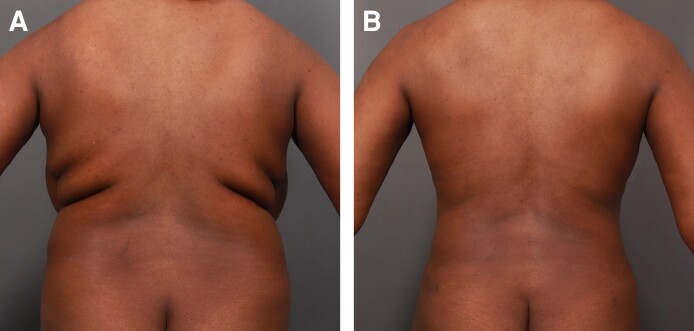
Results from an ultrasonic-assisted liposuction procedure with the utilization of a helium-based plasma system. A 50-year-old female (A) before and (B) after receiving an ultrasonic-assisted liposuction procedure with the utilization of a helium-based plasma system. After image was taken 10 months postoperatively.

**Figure 3.**
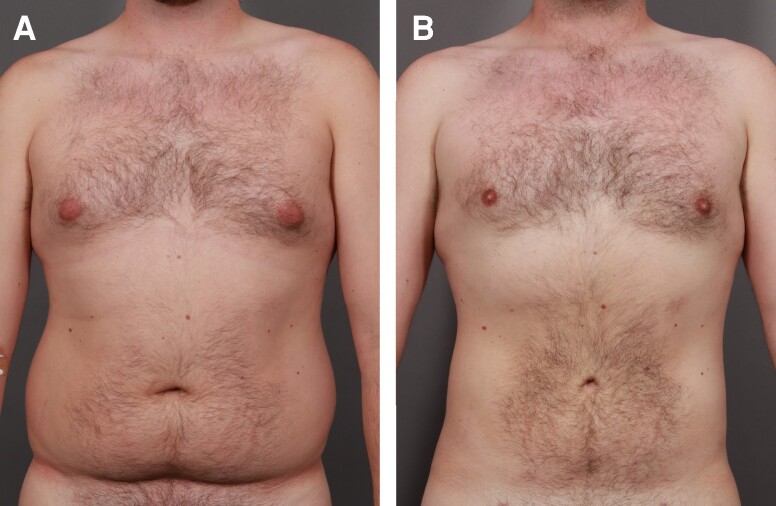
Results from ultrasonic-assisted liposuction procedure without the utilization of a helium-based plasma system. A 25-year-old male (A) before and (B) after receiving an ultrasonic-assisted liposuction procedure without the utilization of a helium-based plasma system. After image was taken 1 year and 5 months postoperatively.

**Figure 4.**
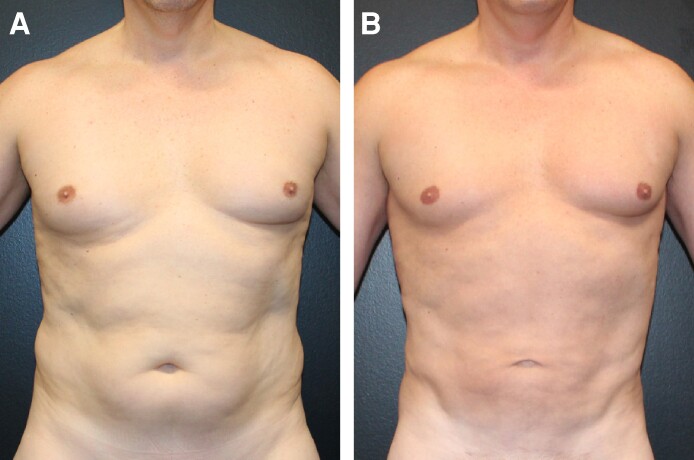
Results from an ultrasonic-assisted liposuction procedure with the utilization of a helium-based plasma system. A 52-year-old male (A) before and (B) after receiving an ultrasonic-assisted liposuction procedure with the utilization of a helium-based plasma system. After image was taken 1 year postoperatively.

Each of the 3 treatment groups (Groups 1-3) consisted of 50 patients. A female patient's results from a UAL procedure without the utilization of a helium-based plasma system (Figure 1) can be compared with results from a UAL procedure with the utilization of a helium-based plasma system (Figure 2). A male patient's results from a UAL procedure without the utilization of a helium-based plasma system (Figure 3) can be compared with results from a UAL procedure with the utilization of a helium-based plasma system (Figure 4).

A 1-way ANOVA demonstrated a significant difference (*P* < .05) in BMI between Groups 1 and 2, *F*(2,87) = 8.6, *P* = .0043. However, there was no significant association in BMI between Groups 1 and 3, or Groups 2 and 3.

Many patients (83.3%, *n* = 125) had multiple (>1) body areas treated with UAL during the same procedure. Most of these patients (65.4%, *n* = 98) underwent UAL performed on 2 to 4 body areas. In Groups 1 and 2, the majority of patients (26%, *n* = 13, and 20%, *n* = 10, respectively) had 3 body areas treated. In Group 3, most patients (26%, *n* = 13) had 4 body areas treated (Table 2).

**Table 2.** Number of Body Areas Treated With Ultrasound-Assisted Liposuction

	Group 1 (*n* = 50)	Group 2 (*n* = 50)	Group 3 (*n* = 50)	Total (*n* = 150)
1	14% (7/50)	18% (9/50)	18% (9/50)	16.7% (25/150)
2	24% (12/50)	16% (8/50)	22% (11/50)	20.7% (31/150)
3	26% (13/50)	20% (10/50)	22% (11/50)	22.7% (34/150)
4	22% (11/50)	18% (9/50)	26% (13/50)	22% (33/150)
5	10% (5/50)	10% (5/50)	6% (3/50)	8.7% (13/150)
6	4% (2/50)	16% (8/50)	6% (3/50)	8.7% (13/150)
7	0% (0/50)	0% (0/50)	0% (0/50)	0% (0/150)
8	0% (0/50)	2% (1/50)	0% (0/50)	0.7% (1/150)

Of the body areas treated, most patients had a procedure performed on their hips and/or abdomen (65.33%, *n* = 98, and 64%, *n* = 96, respectively). Additional body areas treated were the neck/submental area, thighs, arms, scapular rolls, lower legs, breast/chest, infra-axillary region, buttocks, and midback (Table 3).

**Table 3.** Body Areas Treated by Enrollment Group

	Group 1 (*n* = 50)	Group 2 (*n* = 50)	Group 3 (*n* = 50)	Total patient population (*n* = 150)
Neck/submental area	14% (7/50)	38% (19/50)	18% (9/50)	23.33% (35/150)
Hips or love handles	72% (36/50)	60% (30/50)	64% (32/50)	65.33% (98/150)
Thighs	36% (18/50)	46% (23/50)	42% (21/50)	41.33% (62/150)
Arms	6% (3/50)	28% (14/50)	22% (11/50)	18.67% (28/150)
Scapular rolls	0% (0/50)	2% (1/50)	2% (1/50)	1.33% (2/150)
Lower legs	4% (2/50)	12% (6/50)	2% (1/50)	6% (9/150)
Breast/chest	38% (19/50)	52% (26/50)	88% (14/50)	39.33% (59/150)
Infra-axillary region	22% (11/50)	16% (8/50)	32% (16/50)	23.33% (25/150)
Abdomen	64% (32/50)	60% (30/50)	68% (34/50)	64% (96/150)
Buttocks	18% (9/50)	28% (14/50)	18% (9/50)	21.33% (32/150)
Midback	24% (12/50)	2% (1/50)	0% (0/50)	8.6% (13/150)
Other	4% (2/50)	0% (0/50)	2% (1/50)	2% (3/150)

Out of the total patient population, 72.6% (*n* = 109) had concomitant procedure(s) performed. Among these patients, 29.3% (*n* = 32) were in Group 1, 33.9% (*n* = 37) were in Group 2, and 36.6% (*n* = 40) were in Group 3 (Table 4). The majority of patients (75%, 78%, 60% across all 3 groups, respectively) who had concomitant procedures performed underwent fat injection procedures. Other notable concomitant procedures included abdominoplasty in 31.3% (*n* = 10) of Group 1 and 35% (*n* = 14) of Group 3.

**Table 4.** Breakdown of Concomitant Procedures Performed

	Group 1 (*n* = 32)	Group 2 (*n* = 37)	Group 3 (*n* = 40)	Total patient population who received concomitant procedures (*n* = 109)
Skin excision	0% (0/32)	0% (0/37)	2% (1/40)	0.9% (1/109)
Blepharoplasty	6.3% (2/32)	8% (3/37)	5% (2/40)	6.4% (7/109)
Face lift	3.1% (1/32)	0% (0/37)	0% (0/40)	0.9% (1/109)
Neck lift	6.3% (2/32)	0% (0/37)	2% (1/40)	2.7% (3/109)
CO_2_ resurfacing	0% (0/32)	0% (0/37)	5% (2/40)	1.8% (2/109)
Helium-plasma resurfacing	3.1% (1/32)	8% (3/37)	2% (1/40)	4.5% (5/109)
Implant exchange	6.3% (2/32)	10% (4/37)	7% (3/40)	8.2% (9/109)
Breast reduction/gynecomastia	9.3% (3/32)	8% (3/37)	5% (2/40)	5.5% (6/109)
Breast augmentation	3.1% (1/32)	8% (3/37)	5% (2/40)	5.5% (6/109)
Mastopexy	15.6% (5/32)	13% (5/37)	25% (10/40)	18.3% (20/109)
Abdominoplasty	31.3% (10/32)	8% (3/37)	35% (14/40)	24.7% (27/109)
Brazilian buttock lift	9.4% (3/32)	13% (5/37)	2% (1/40)	8.2% (9/109)
Fat injection	75% (24/32)	78% (29/37)	60% (24/40)	70.6% (77/109)
Other	31.3% (10/32)	5.4% (2/37)	5% (2/40)	12.8% (14/109)

Other includes: Scar Revision, Pectoral Implants, Rhinoplasty, Genioplasty, and Brachioplasty procedures.

Treatment settings data were collected, when available, for patients who underwent a UAL procedure with the adjunct utilization of the helium-based plasma system (Tables 5, 6). Most patients (46.3%, *n* = 19) in Group 2 were treated with a helium setting of 4 LPM, and most patients (82.2%, *n* = 37) in Group 3 were treated with 2 LPM. The majority of patients in both groups (60% and 73.3%, respectively) were treated with 27 cm handpieces. In Group 2, most patients (37%, *n* = 10) received 3 to 4 passes in documented treatment areas. In Group 3, most patients (60%) received passes in documented treatment areas.

**Table 5.** Helium-Based Plasma Procedure Details (When Available)

	Group 2 (*n* = 50)	Group 3 (*n* = 50)	Total helium-based plasma (*n* = 100)
Helium setting (LPM)	(*n* = 41)	(*n* = 45)	(*n* = 86)
1.5	7.3% (3/41)	15.6% (7/45)	11.6% (10/86)
2	4.9% (2/41)	82.2% (37/45)	45.3% (39/86)
2.5	14.6% (6/41)	0.0% (0/45)	7.0% (6/86)
3	24.4% (10/41)	0.0% (0/45)	11.6% (10/86)
3.5	2.4% (1/41)	0.0% (0/45)	1.2% (1/86)
4	46.3% (19/41)	2.2% (1/45)	23.3% (20/86)
Handpiece size (cm)	(*n* = 4)	(*n* = 15)	(*n* = 19)
15	25% (1/4)	20% (3/15)	21% (4/19)
27	75% (3/4)	80% (12/15)	78.9% (15/19)
No. of passes	(*n* = 27)	(*n* = 5)	(*n* = 32)
2	3.7% (1/27)	0% (0/5)	3.1% (1/32)
3	18.5% (5/27)	0% (0/5)	15.6% (5/32)
“3 to 5”	3.7% (1/27)	0% (0/5)	3.1% (1/32)
4	18.5% (5/27)	0% (0/5)	15.6% (5/32)
“4 to 5”	3.7% (1/27)	0% (0/5)	3.1% (1/32)
5	3.7% (1/27)	20% (1/5)	6.3% (2/32)
6	0% (0/27)	60% (3/5)	9.4% (3/32)
7	0% (0/27)	20% (1/5)	3.1% (1/32)

**Table 6.** Helium-Based Plasma Procedure Details Continued-Power Settings (When Available)

	Group 2 (*n* = 50)	Group 3 (*n* = 50)	Total helium-based plasma (*n* = 100)
Neck/submental			
60%	11.1% (2/18)	0% (0/19)	5.4% (2/37)
65%	11.1% (2/18)	0% (0/19)	5.4% (2/37)
70%	16.7% (3/18)	10.5% (2/19)	13.5% (5/37)
75%	5.6% (1/18)	31.6% (6/19)	18.9% (7/37)
80%	22.2% (6/18)	57.9% (11/19)	40.5% (15/37
85%	33.3% (6/18)	0% (0/19)	16.2 (6/37)
Hips			
60%	4.2% (1/24)	0% (0/9)	3% (1/33)
70%	25% (6/24)	0% (0/9)	18.2% (6/33)
75%	0% (0/24)	33.3% (3/9)	9.1% (3/33)
80%	25% (6/24)	66.7% (6/9)	36.4% (12/33)
85%	45.8% (11/24)	0% (0/9)	33.3% (11/33)
Thighs			
60%	5.6% (1/18)	0% (0/1)	5.3% (1/19)
70%	27.8% (5/18)	0% (0/1)	26.3% (5/19)
80%	27.8% (5/18)	100% (1/1)	31.6% (6/19)
85%	38.9% (7/18)	0% (0/1)	36.8% (7/19)
Arms			
70%	40% (4/10)	0% (0/3)	30.8% (4/13)
75%	0% (0/10)	100% (3/3)	23.1% (3/13)
80%	30% (3/10)	0% (0/3)	23.1% (3/13)
85%	30% (3/10)	0% (0/3)	23.1% (3/13)
Scapular rolls			
70%	50% (1/2)	0% (0/12)	7.1% (1/14)
75%	50% (1/2)	41.7% (5/12)	42.9% (6/14)
80%	0% (0/2)	58.3% (7/12)	50% (7/14)
Lower legs			
70%	50% (2/4)	8.3% (1/12)	18.8% (3/16)
75%	0% (0/4)	33.3% (4/12)	25% (4/16)
80%	25% (1/4)	58.3% (7/12)	50% (8/16)
85%	25% (1/4)	0% (0/12)	6.3% (1/16)
Breast/chest			
60%	5.6% (1/18)	0% (0/27)	2.2% (1/45)
65%	5.6% (1/18)	0% (0/27)	2.2% (1/45)
70%	16.7% (3/18)	7.4% (2/27)	11.1% (5/45)
75%	5.6% (1/18)	33.3% (9/27)	22.2% (10/45)
80%	38.9% (7/18)	59.3% (16/27)	51.1% (23/45)
85%	27.8% (5/18)	0% (0/27)	11.1% (5/45)
Infra-axillary			
65%	0% (0/7)	14.3% (1/7)	7.1% (1/14)
70%	42.9% (3/7)	0% (0/7)	21.4% (3/14)
75%	14.3% (1/7)	42.9% (3/7)	28.6% (4/14)
80%	14.3% (1/7)	42.9% (3/7)	28.6% (4/14)
85%	28.6% (2/7)	0% (0/7)	14.3% (2/14)
Abdomen			
60%	4.2% (1/24)	0% (0/2)	3.8% (1/26)
70%	29.2% (7/24)	0% (0/2)	26.9% (7/26)
75%	0% (0/24)	100% (2/2)	7.7% (2/26)
80%	25% (6/24)	0% (0/2)	23.1% (6/26)
85%	41.7% (10/24)	0% (0/2)	38.5% (10/26
Buttock			
60%	10% (1/10)	0% (0/6)	6.3% (1/16)
70%	40% (4/10)	0% (0/6)	25% (4/16)
75%	10% (1/10)	66.7% (4/6)	31.3% (5/16)
80%	20% (2/10)	33.3% (2/6)	25% (4/16)
85%	20% (2/10)	0% (0/6)	12.5% (2/16)
Midback			
70%	100% (1/1)	0% (0/2)	33.3% (1/3)
75%	0% (0/1)	100% (2/2)	66.7% (2/3)

Other treatment areas excluded from the table were Mons Pubis with 100% (1/1) treatment of the Mons Pubis in Group 3.

All patients were seen per site standard of care for posttreatment follow-up. The mean patient follow-up time after treatment was 10.2 months (range, 6 months to 2 years) overall and 10.5, 12, and 8 months for Groups 1 to 3, respectively, postprocedure.

A total of 301 instances of expected treatment effects among 134 patients (89.3% of total patients) were reported (Table 7). The expected treatment effects, or typical treatment side effects, of the UAL and/or helium-plasma device of mild-to-moderate severity and lasting up to a typical maximum duration, were bruising/ecchymosis, crepitus, edema, emesis, erythema, fever, hypoesthesia/numbness, pain, nausea, pruritus/itching, hyperpigmentation, hypopigmentation, and urticaria. Of those events, 31.6% (*n* = 95) were in Group 1, 37.2% (*n* = 112) were in Group 2, and 30.9% (*n* = 93) were in Group 3 (Table 7).

**Table 7.** Summary of Occurring Expected Treatment Effects

	Group 1 (*n* = 50)	Group 2 (*n* = 50)	Group 3 (*n* = 50)	Total helium-based plasma (*n* = 100)
Bruise/ecchymosis	22.0% (11/50)	38.0% (19/50)	26.0% (13/50)	32% (32/100)
Crepitus	0.0% (0/50)	2.0% (1/50)	0.0% (0/50)	1.0% (1/100)
Edema	64.0% (32/50)	76.0% (38/50)	72.0% (36/50)	74.0% (74/100)
Emesis	4.0% (2/50)	4.0% (2/50)	0.0% (0/50)	2.0% (2/100)
Erythema	12.0% (6/50)	2.0% (1/50)	4.0% (2/50)	3.0% (3/100)
Fever	2.0% (1/50)	0.0% (0/50)	2.0% (1/50)	1.0% (1/100)
Hypoesthesia/numbness	0.0% (0/50)	0.0% (0/50)	2.0% (1/50)	1.0% (1/100)
Pain	60.0% (30/50)	82.0% (41/50)	56.0% (28/50)	69.0% (69/100)
Nausea	20.0% (10/50)	14.0% (7/50)	14.0% (7/50)	14.0% (14/100)
Pruritus/itching	0.0% (0/50)	2.0% (1/50)	4.0% (2/50)	3.0% (3/100)
Hyperpigmentation	4.0% (2/50)	6.0% (3/50)	2.0% (1/50)	4.0% (4/100)
Hypopigmentation	2.0% (1/50)	0.0% (0/50)	0.0% (0/50)	0.0% (0/100)
Urticaria	0.0% (0/50)	0.0% (0/50)	4.0% (2/50)	2.0% (2/100)

A χ^2^ test of independence was conducted to examine the relationship between treatment groups and occurring expected treatment effects. There was a significant relationship (*P* < .05) between “Pain” and all 3 groups *X*(2, *N* = 150) = 8.7, *P* = .013. Further analysis showed the proportion of patients who reported Pain increased from Group 1 to Group 2, *X*(1, *N* = 100) = 7.90, *P* = .0049. There was no significant association between all other variables and the treatment groups.

There were no serious AEs or unexpected AEs reported (Table 8). A total of 73 instances of AEs among 72 patients (48% of total patients) were reported. The AEs, or new medical problems/exacerbation of an existing problem experienced by a patient postoperatively whether or not considered to be procedure and/or device related by the investigator, were hematoma, hypertrophic scarring, infection, seroma, lesion, visual disturbances, wound, pneumonia, and “Other.” The Other category consisted of patient dissatisfaction, constipation, urinary retention, penile swelling, scrotal swelling, emergency room visit, back pain, night sweats, limited range of motion, tachycardia, anxiety, and residual skin and contour laxity. Of the AEs, 39.7% (*n* = 29) were in Group 1, 34.2% (*n* = 25) were in Group 2, and 26% (*n* = 19) were in Group 3 (Table 8).

**Table 8.** Summary of Occurring Adverse Events

	Group 1 (*n* = 50)	Group 2 (*n* = 50)	Group 3 (*n* = 50)	Total helium-based plasma (*n* = 100)
Any serious AE	0.0% (0/50)	0.0% (0/50)	0.0% (0/50)	0.0% (0/100)
Hematoma	2.0% (1/50)	4.0% (2/50)	0.0% (0/50)	2.0% (2/100)
Hypertrophic scarring	2.0% (1/50)	2.0% (1/50)	6.0% (3/50)	4.0% (4/100)
Infection	4.0% (2/50)	0.0% (0/50)	4.0% (2/50)	2.0% (2/100)
Seroma	14.0% (7/50)	14.0% (7/50)	12.0% (6/50)	13.0% (13/100)
Lesion	2.0% (1/50)	0.0% (0/50)	0.0% (0/50)	0.0% (0/100)
Visual disturbances	0.0% (0/50)	2.0% (1/50)	0.0% (0/50)	1.0% (1/100)
Wound	8.0% (4/50)	10.0% (5/50)	10.0% (5/50)	10.0% (10/100)
Pneumonia	2.0% (1/50)	0.0% (0/50)	0.0% (0/50)	0.0% (0/100)
Other	24.0% (12/50)	18.0% (9/50)	6.0% (3/50)	12.0% (12/100)

AE, adverse event.

A χ^2^ test of independence was conducted to examine the relationship between treatment groups and occurring AEs. There was a significant relationship (*P* < .05) between treatment groups and Other AEs, *X*(2, *N* = 150) = 6.25, *P* = .0439. The AE category Other includes 36 different events (eg, urinary retention, constipation, penile swelling, lightheadedness, back pain, patient dissatisfaction, weakness, insomnia, tachycardia, anxiety, capsular contraction) that occurred infrequently enough (<10 reported instances) to be grouped as Other for our analysis.

Patients with reported AEs and/or expected treatment effects were treated per the site's standard of care and seen for routine follow-up. Approximately 88% (*n* = 44) of the patients in Group 1 and 92% (*n* = 46) of the patients in Groups 2 and 3 were prescribed a form of pain medication postoperatively. The majority (68%) of reported pain events were reported to be “general” rather than localized to any specific body areas. All reported AEs resolved before the last documented follow-up appointment. Relatedness of AEs was not found documented in the chart review and therefore not included in the analyses.

## DISCUSSION

The field of medicine is ever evolving. With continued practice and advances in the technological space, surgeons have available to them an increasing selection of tools to incorporate as new techniques or in conjunction with current procedures in an effort to improve safety, efficacy, and outcomes. Practitioners rely on data to inform their decisions regarding the utility of new technology within their practices. Few quality studies exist to support these decisions other than short case series. A recent report looking at a sequential experience with transcatheter aortic valve replacement noted a significant improvement in procedural efficiency and safety through a series of 3 sequential 100 case subsets during the adoption of a new technique.^9^ This indirectly supports the findings of the current study suggesting that the observed decrease in AEs and improved outcomes in Group 3 are consistent with broader trends seen in medical practice. Furthermore, another review of sequential high-definition liposuction cases noted a dramatic reduction in overall complications from the first 50 cases from 28% to 3%.^10^ The purpose of this study was to evaluate the safety and procedural information for UAL procedures performed by the corresponding author with or without the adjunct utilization of the novel helium-based plasma system and its impact on the learning curve with respect to AEs. The primary surgeon has performed an average of 148 liposuction cases annually over the last 15 years of his career, all primarily ultrasound assisted. The addition of helium-based plasma added the potential for improved skin retraction through the unique targeted coupling and precision heating through selective impedance, targeting the collagen of the fibroseptal network in the subdermal space. A chart review of 150 male and female patients from a single site was conducted.

The UAL portion of these treatments remained relatively similar across the 3 patient groups with most patients (83.3%) undergoing treatment to multiple body areas during the same procedure. Also, the number of concomitant procedures performed was similar among the 3 groups ranging between 32 and 40 patients in each group.

Slight differences were seen in the Renuvion procedure details between Groups 2 and 3. Most patients in Group 2 underwent treatment with 4 LPM of helium administered and 3 to 4 passes per treatment area, whereas the majority of Group 3 underwent treatment with 2 LPM and 6 passes. Accumulated data regarding energy distribution, helium flow rates, temperature curves, pace of treatment probe, and probe design advancements account for the treatment parameter differences between Groups 2 and 3.

With the similarities between UAL treatments and the adjustments made between the groups where the helium-based plasma was utilized, we saw improvement in safety in Group 3 compared with the other 2 groups. The number of reported AEs rose from 124 AEs in Group 1 to 138 AEs and ETEs in Group 2, as expected on the introduction of a new surgical device to a procedure. After continued use and observation of the device, the number of reported AEs and ETEs decreased to 112 events in Group 3, showing the least number of reported AEs and ETEs across all 3 patient groups.

The χ^2^ test of independence conducted to examine the relationship between treatment groups and expected treatment effects found a significant relationship (*P* < .05) between Pain and all 3 groups. Further analysis showed the proportion of patients who reported Pain increased from Group 1 to Group 2, whereas the proportion of patients who reported Pain decreased from Group 2 to Group 3. The data indicate that the implementation of helium-based plasma as an adjunct procedure to UAL treatments initially resulted in an increase in patient-reported pain (60% in Group 1 to 82% in Group 2); however, as the surgeon became more proficient with the device, patient-reported pain decreased (56% in Group 3) compared with both Group 1 and Group 2, demonstrating the learning-curve aspect of this study.

A significant (*P* < .05) relationship was found between treatment groups and Other AEs after running a χ^2^ test of independence on the data presented in Table 8. This is the only statistically significant (*P* < .05) association found for AEs likely because various side effects were included in 1 category. The Other category included AEs that had an occurrence of <10 reported instances illustrating that they were isolated events.

No serious AEs (SAEs) were observed or reported. The 2 most common events reported were “Edema,” in 70% of patients treated, and Pain in 66% of patients treated. All patients experiencing AEs were treated per the site's standard of care and seen for routine follow-up appointments. All reported AEs in this study are a known and expected risk of both UAL and the adjunct utilization of the helium-based plasma system. Although these AEs occurred in patients who were treated with or without helium-based plasma in conjunction with UAL, they are also a known and expected risk for subdermal procedures utilizing tumescent anesthesia and/or undermining of soft tissue. Of note was the consistency of the seroma rate across all treatment groups. This would indicate that the seroma rate is more likely associated with liposuction rather than an additional energy source.^11^

The data demonstrate that the helium-based plasma system was implemented into the corresponding authors’ practice without compromising patient safety and that, after continued use and observation (the learning curve), safety improved in comparison with procedures before the implementation of the system. Additionally, in the corresponding author's experience and based on their clinical evaluation, they saw an increase in the level of skin contraction achieved utilizing the helium-based plasma system as an adjunct procedure to UAL treatment compared with the level of skin contraction achieved with UAL solely.

This study is limited due to the retrospective design and data availability during chart review, lack of objective measures for skin contractility, and the data being from 1 experienced operator at a single site. Additional retrospective research, including documented patient satisfaction rates and objective measures of skin contraction, and additional prospective research involving multisite and multi-investigator studies, are needed to evaluate the safety and efficacy of adjunct utilization of the helium-based plasma system during UAL procedures.

## CONCLUSIONS

The purpose of this study was to evaluate the impact of a learning curve on safety and procedural information for UAL procedures with or without the adjunct utilization of the helium-based plasma system. A review of 150 male and female patient charts from a single site was conducted. Study data support the positive impact of progressive experience on safety when using the helium-based plasma system as an adjunct procedure during UAL procedures. Additional prospective research is needed to further improve the understanding of how progressive experience impacts endpoints, such as intraoperative surgical efficiency, return to normal daily activity, return to work, and patient satisfaction.
